# Metallic Mercury as a Purgative

**Published:** 1902-05-03

**Authors:** 


### METALLIC MERCURY AS A PURGATIVE.

We are all inclined to laugh at ancient remedies, partly, no doubt, because we see no reason in their action, whereas, even the dullest of us can follow the working of the surgeon's knife. There is something so definite and positive, for example, in the treatment of intestinal obstruction by operation that one hardly has patience to listen to such an antiquated suggestion as that of loading the man's stomach up with mercury and leaving this weighty fluid to shake itself down as best it may. Yet we find a report this week of two cases, evidently very serious cases of obstruction, in which this "remedy" was tried, and in both of which, to say the very least of it, recovery followed the treatment. The cases are recorded by Dr. McKean Harrison.[1] The first was a man, aged 60, who, the day after a fall, complained of pain in the umbilical region which increased day by day. The bowels were not moved, notwithstanding the use of various aperients and enemata. Operation was declined, and at the end of nine days his condition was most serious, vomiting, hiccough, and great distension being present. Half a pound of mercury was then administered, and a grain of opium was given every four hours. Next day he was easier, and the day after that the bowels were moved. He recovered. The second case was even worse. He was 80 years old, and was suddenly attacked with severe pain in the epigastric region. The second day he vomited blood. At the end of three days he was in a desperate condition, pulse quick and running, tongue dry, constant sickness, delirium, and great abdominal distension. Gangrene was feared; yet half a pound of mercury was administered, and opium pills every four hours. The next morning the bowels were moved. This patient also got well. Now what the moral of these cases is we will not presume to say. Whether they were illustrations of the old fallacy, *post hoc procter hoc*, whether it was the opium that did the good, whether it was that just as all hope was being given up the remedies which had already been administered began to act, or whether really it was the mercury that did the good, sagging and pulling, and by sheer weight disentangling the overlying loop of gut, or breaking up the impacted fæces, none of us probably can tell. But they are interesting cases, although it is to be hoped that no one will be tempted by their satisfactory issue to postpone surgical intervention when it is demanded.

[1] Brit. Med. Jour., April 26.

